# Impaired Regulation by IL-35 in Systemic Sclerosis

**DOI:** 10.3390/ijms241310567

**Published:** 2023-06-24

**Authors:** Rubén Osuna-Gómez, Ivan Castellví, Maria Mulet, Mª Àngels Ortiz, Douglas E. Brough, Helen Sabzevari, Roshanak T. Semnani, Silvia Vidal

**Affiliations:** 1 Inflammatory Diseases, Biomedical Research Institute Sant Pau (IIB Sant Pau), 08041 Barcelona, Spain; rosuna@santpau.cat (R.O.-G.); mmulet@santpau.cat (M.M.); mortiz@santpau.cat (M.À.O.); 2 Department of Rheumatology and Systemic Autoimmune Diseases, Hospital de la Santa Creu i Sant Pau, 08041 Barcelona, Spain; icastellvi@santpau.cat; 3 Precigen, Inc., Germantown, MD 20876, USA; dbrough@precigen.com (D.E.B.); hsabzevari@precigen.com (H.S.); rsemnani@precigen.com (R.T.S.)

**Keywords:** systemic sclerosis, interleukin-35, CD4+ T lymphocytes, regulatory T cells

## Abstract

This study investigated the role of IL-35 in systemic sclerosis (SSc) patients, focusing on CD4+ T cell response and immunomodulatory cytokine production. By comparing the cytokine levels in healthy donors (HD) and SSc patients using ELISAs, we found a significantly lower plasma IL-35 concentration in the SSc patients (52.1 ± 5.6 vs. 143 ± 11.1, *p* < 0.001). Notably, the IL-35 levels showed a negative correlation with TGF-β (*p* < 0.001) and IL-17 (*p* = 0.04). Assessing the IL-35R expression across cell types in the SSc patients and HDs via flow cytometry, we found higher levels on monocytes (40.7 + 5.7 vs. 20.3 ± 1.9, *p* < 0.001) and lower levels on CD8+ T cells (61.8 ± 9.2 vs. 83.4 ± 0.8, *p* < 0.05) in the SSc patients. The addition of recombinant IL-35 to stimulated peripheral blood mononuclear cells reduced the IL-17+CD4+ T cell percentage (9.0 ± 1.5 vs. 4.8 ± 0.7, *p* < 0.05) and increased the IL-35+CD4+ T percentage (4.1 ± 2.3 vs. 10.2 ± 0.8, *p* < 0.001). In a Treg:Tresponder cell Sco-culture assay with HD and SSc samples, rIL35 decreased the cell proliferation and levels of IL-17A (178.2 ± 30.5 pg/mL vs. 37.4 ± 6.4 pg/mL, *p* < 0.001) and TGF-β (4194 ± 777 pg/mL vs. 2413 ± 608 pg/mL, *p* < 0.01). Furthermore, we observed a positive correlation between the modified Rodnan skin score (mRSS) and TGF-β (*p* < 0.001), while there was a negative correlation between mRSS and IL-35 (*p* = 0.004). Interestingly, higher levels of plasmatic IL-35 were detected in individuals with limited disease compared to those with diffuse disease (60.1 ± 8.0 vs. 832.3 ± 4.1, *p* < 0.05). These findings suggest that IL-35 exhibits anti-inflammatory properties in SSc and it may serve as a marker for disease severity and a therapeutic target.

## 1. Introduction

Systemic sclerosis (SSc) is a connective disease with no fully understood pathogenesis. Its contributing factors are pro-inflammatory cytokines, the tissue microenvironment, and inflammation [1,2]. The excessive activation of T cells in SSc patients seems to be closely involved in their vascular disease, fibrosis, and humoral immunity [3]. Up- and down-regulated cell surface molecules, the altered release of pro-fibrotic and pro-inflammatory cytokines, and the direct contact of endothelial cells and fibroblasts are some of the cellular abnormalities associated with the pathogenesis of SSc [4]. As such, the levels of T follicular helper (Tfh) cells, regulatory T sells (Tregs), CD4+ cytotoxic T lymphocytes (CTLs), angiogenic T (Tang) cells, and Th2-related (IL-4, IL-10, IL-13) or Th17-related (IL-6, IL-17A) cytokines are higher in the plasma of patients than controls [5,6,7,8]. These abnormalities induce a Th1/Th2 and Th17/Treg imbalance in SSc, which may be closely related to the abnormal immune status of SSc patients [8,9].

Tregs play a central role in preventing autoimmune disease due to their suppressive functions, which include the production of inhibitory cytokines such as IL-10, TGF-β, and IL-35 [10,11,12]. The effect of Tregs in SSc is not well elucidated. Current research has shown that Treg cells participate in SSc through contact-dependent cell suppression and inhibitory cytokine release mechanisms [13]. Despite SSc patients having an increased number of Tregs, they have decreased immunosuppressive cytokine release and a reduced ability to suppress effector T cells [14,15,16].

IL-35 is a member of the IL-12 family, composed of p35 (IL-12A) and Ebi3 (Epstein–Barr virus-induced gene 3) subunits [17]. This cytokine, which is mainly secreted by Tregs, has strong suppressive properties, both in vivo and in vitro [11]. Not surprisingly, Tregs from specific knockouts of Ebi3 or p35 mice have decreased this suppressive activity [18]. Compared to IL-35, the pleiotropic cytokines IL-10 and TGF-β are produced primarily by monocytes and T cells with suppressive functions [19]. Recent reports have shown that different Treg signaling could generate distinct Treg effector subsets with complementary roles in the maintenance of tolerance. Thus, a differential TCR signal strength generates distinct effector IL-10+ and IL-35+ Treg cell subsets with synergistic functions for controlling autoimmunity [20]. In line with IL-35 mechanisms, its levels are lower in patients with active autoimmunity disease (systemic lupus erythematosus, rheumatoid arthritis, and autoimmune interstitial lung disease) than those with inactive disease [21,22,23].

To determine the role of IL-35 in SSc, we first measured the levels of IL-35 and pro-inflammatory cytokines in the plasma of patients. We then studied the association of the concentrations of IL-35 with pro-inflammatory cytokine and peripheral blood mononuclear cell (PBMC) subpopulations. In addition, we analyzed the regulatory effects of recombinant IL-35 (rIl-35) on PBMCs using in vitro activation assays and co-cultures of T responder cells (Tres) with T regulatory cells (Tregs). Finally, we established an association between IL-35 and TGF-β with clinical parameters and SSc activity.

## 2. Results

### 2.1. Population

Thirty-two patients were included in the study. A summary of the patients’ characteristics is given in Table 1. The mean age was 65 years, with a predominance of females (31 cases). Eight cases (25%) were classified as limited and twenty-four cases (75%) as diffuse SSc. The most frequent symptom was skin/vascular, followed by gastrointestinal and pulmonary involvement.

### 2.2. Plasma Levels of IL-35 and Association with Circulating Subsets of Leukocytes

**Figure 1:**
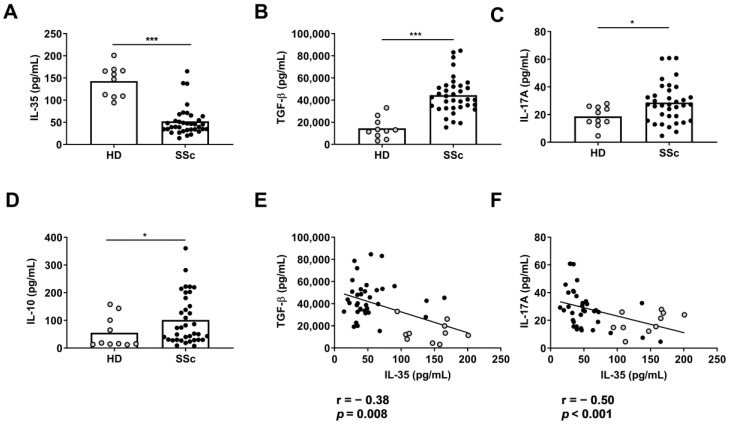
Plasma levels of (**A**) IL-35, (**B**) TGF-β, (**C**) IL-17A, and (**D**) IL-10 cytokines determined using ELISA in HDs (n = 10) and SSc (n = 37) patients (unpaired *t*-test and Mann–Whitney U test). Correlation between plasma levels of IL-35 with (**E**) TGF-β or (**F**) IL-17A from both groups (Spearman correlation). Spots in black correspond to SSc patients and spots in gray to HDs. * *p* < 0.05; *** *p* < 0.001.

We first compared the plasma levels of IL-35, TGF-β, IL-17, and IL-10 in healthy donors (HD) and SSc patients using ELISAs. The IL-35 levels were significantly lower in the SSc patients than the HDs (*p* < 0.001) (Figure 1A). In contrast, the plasma TGF-β, IL-17, and IL-10 levels were significantly higher in the SSc patients than the HDs (Figure 1B–D). Furthermore, we found a negative correlation between the IL-35 and TGF-β levels and between the IL-17A and IL-35 levels in all the groups (Figure 1E,F).

**Figure 2:**
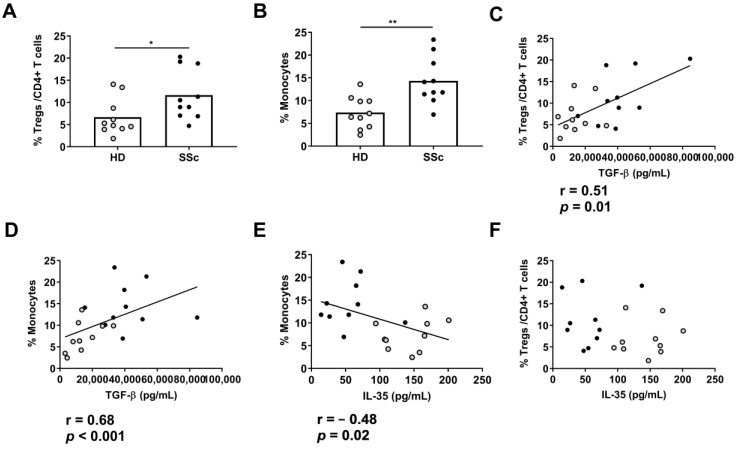
Percentage of (**A**) Tregs and (**B**) monocytes in PBMCS from HDs (n = 10) and SSc (n = 10) patients (unpaired *t*-test and Mann–Whitney U test). Correlation between plasma levels of TGF-β with (**C**) the percentage of Tregs or (**D**) of monocytes from both groups (Pearson and Spearman correlation). Correlation between plasma levels of IL-35 with (**E**) the percentage of Tregs or (**F**) of monocytes from both groups (Pearson and Spearman correlation). Spots in black correspond to SSc patients and spots in gray to HDs. * *p* < 0.05; and ** *p* < 0.01.

We found that the percentages of Tregs and monocytes were higher in the SSc patients than the HDs (*p* < 0.05 and *p* < 0.01, respectively) (Figure 2A,B), without differences in the percentages of CD4+ T cells, CD8+ T cells, B cells, and NK cells (data not shown). Furthermore, the plasmatic TGF-β levels were correlated with the percentages of Tregs and monocytes in all the groups (Figure 2C,D). Moreover, we observed a negative correlation between the percentage of monocytes, but not Tregs, and the IL-35 plasma levels in all the groups (Figure 2E,F).

### 2.3. Expression of IL35R on PBMCs

**Figure 3:**
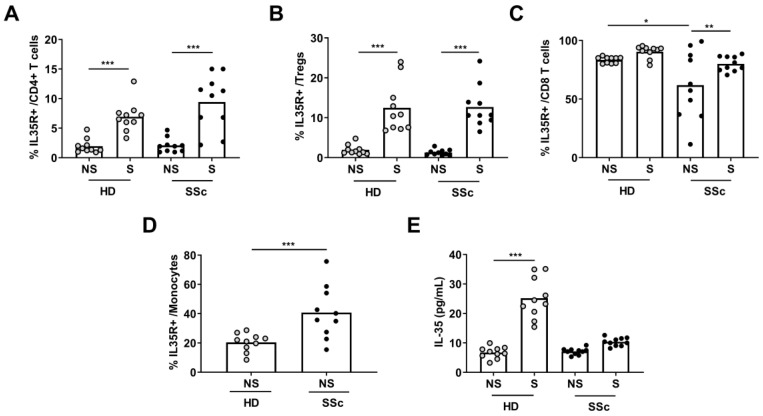
Determination of IL-35 receptor. Peripheral blood mononuclear cells (PBMCs) from HDs (n = 10) or SSc (n = 10) patients were cultured with (S) or without (NS) activation cocktail (an-ti-CD3/anti-CD28) for 72 h. After gating each subset, the expression of the corresponding IL-35 receptor (gp130+ IL-12Rβ2+) was determined on (**A**) CD4+ T cells, (**B**) Tregs, and (**C**) CD8+ T cells (paired *t*-test and Mann–Whitney U test). (**D**) Determination of IL-35 receptor on monocytes was evaluated only in NS conditions (Mann–Whitney U test). (**E**) Levels of IL-35 in culture supernatant were evaluated using ELISA (paired *t*-test). Spots in black correspond to SSc patients and spots in gray to HDs. * *p* < 0.05; ** *p* < 0.01; and *** *p* < 0.001.

To determine whether PBMC stimulation can induce the upregulation of IL-35 receptors (IL-35R), we compared the PBMCs from HDs and SSc patients cultivated with or without a T cell activator. We found that the percentages of IL-35R+ on CD4+ T cells and Tregs were similar in the cultures of cells from both groups, with and without activation (Figure 3A,B). However, the percentage of IL-35R+ on CD8+ T cells was lower in the SSc patients than the HDs in non-stimulated conditions (*p* < 0.05) and similar in both groups with activation (Figure 3C). In contrast, the percentage of IL-35R+ in monocytes was higher in the SSc patients than the HDs without activation (*p* < 0.001) (Figure 3D). Additionally, the IL-35 levels in the supernatant of both groups were similar without activation. However, the IL-35 levels in the supernatant were lower in the SSc patients than the HDs in stimulated conditions (Figure 3E).

### 2.4. Effect of rIl-35 on Intracellular Cytokine Production by CD4+ T Cells

**Figure 4:**
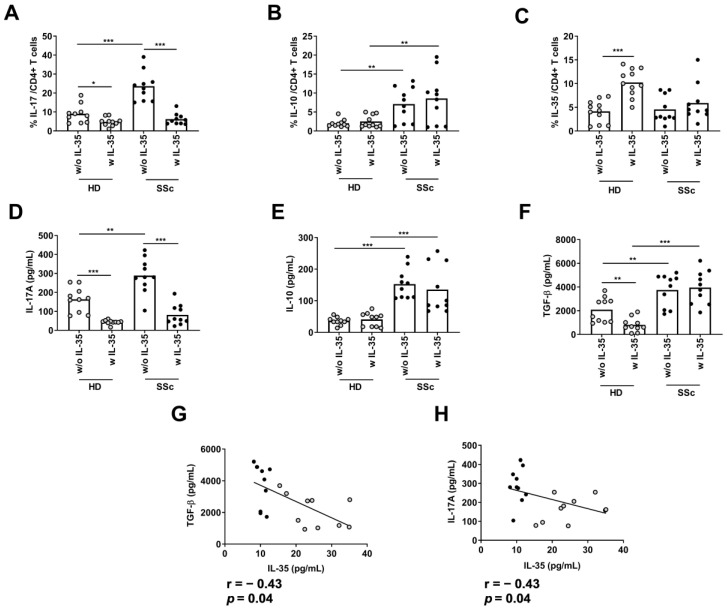
Analysis of the regulatory effect of IL-35 on IL-17, IL-10, TGF-β, and IL-35 secretion. Peripheral blood mononuclear cells (PBMCs) from HDs (n = 10) or SSc patients (n = 10) were cultured with an activation cocktail (anti-CD3/anti-CD28) in the presence or absence of IL-35 for 72 h. After incubation, PBMCs were restimulated with PMA and Ionomicyn over six hours. The intracellular percentages of (**A**) IL-17A-secreting CD4+ T cells, (**B**) IL-10-secreting CD4+ T cells and (**C**) IL-35-secreting CD4+ T cells were measured using flow cytometry (Mann–Whitney U test and Wilcoxon test). Levels of (**D**) IL-17A, (**E**) IL-10, and (**F**) TGF-β on cell culture supernatant were evaluated using ELISAs (Mann–Whitney U test and Wilcoxon test). The correlation between IL-35 and (**G**) TGF-β or (**H**) IL-17A on cell culture supernatants from both groups (Spearman correlation). Spots in black correspond to SSc patients and the spots in gray to HDs. * *p* < 0.05; ** *p* < 0.01; and *** *p* < 0.001.

To analyze the influence of IL-35 on the production of IL-17, IL-10, and IL-35, we activated PBMCs from the HDs and SSc patients in the absence and presence of rIL-35. We found that the percentage of IL-17+CD25+CD4+ T cells was higher in the SSc patients than the HDs in cultures without rIL-35 (*p* < 0.001). However, the percentage of IL-17+CD25+CD4+ T cells decreased in both groups in cultures with rIL-35 (Figure 4A). In contrast, the percentage of IL-10+CD25+CD4+ T cells was higher in the SSc patients than the HDs in cultures without rIL-35. Furthermore, the percentage of IL-10+CD25+CD4+ T cells was similar in both groups in cultures with rIL-35 (Figure 4B). Moreover, the percentage of IL-35+CD25+CD4+ T cells was similar in both groups in cultures without rIL-35. However, the percentage of IL-35+CD25+CD4+ T cells increased when the HD cells (*p* < 0.001), but not SSC cells, were in cultures with rIL-35 (Figure 4C).

In addition, the IL-17A levels on the supernatant were higher in the SSc patients than the HDs in cultures without rIL-35 (*p* < 0.01). However, the IL-17A levels on the supernatants decreased in both groups in cultures with rIL-35 (Figure 4D). In contrast, the IL-10 levels on the supernatant were higher in the SSc patients than the HDs in cultures without rIL-35. Furthermore, the IL-10 levels on the supernatants were similar in both groups in cultures with rIL-35 (Figure 4E). Moreover, the TGF-β levels on the supernatant were higher in the SSc patients than the HDs when the cells were in cultures without rIL-35 (*p* < 0.01). However, the TGF-β levels on the supernatant decreased when the HD cells, but not SSC cells, were in cultures with rIL-35 (*p* < 0.01) (Figure 4F). Furthermore, we found that IL-35 was negatively correlated with the TGF-β and IL-17A levels in the supernatants from cells cultured without rIL-35 in all the groups (Figure 4G–H).

### 2.5. Effect of rIl-35 on Suppression Treg Assay

**Figure 5:**
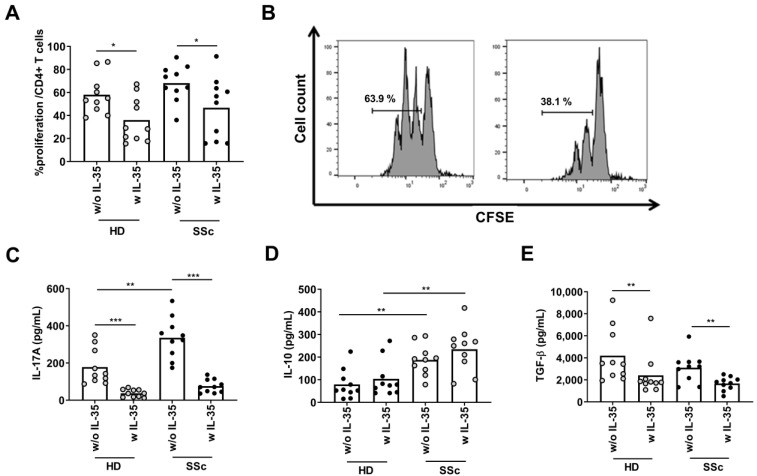
Analysis of the regulatory effect of IL-35 on Treg suppression. Activated T cells (CD4+ CD127+ CD25+) from HDs (n = 10) or SSc patients (n = 10) were incubated with 7 µM of CFSE. CFSE-labelled T cells (Tres) were co-cultured with T regulatory cells (CD4+ CD127- CD25+) (Treg) (Tres:Treg ratio 3:1) with activation cocktail (anti-CD3/anti-CD28) in the presence or absence of IL-35. After five days of culture, proliferation was assessed by measuring the CFSE signals using flow cytometry. (**A**) Proliferation of Tres with their (**B**) histogram representation (Mann–Whitney U test and Wilcoxon test). Levels of (**C**) IL-17A, (**D**) IL-10, and (**E**) TGF-β on cell culture supernatant were evaluated using ELISAs. Spots in black correspond to SSc patients and spots in gray to HDs (Mann–Whitney U test and Wilcoxon test). * *p* < 0.05; ** *p* < 0.01; and *** *p* < 0.001.

To determine the effect of IL-35 on Treg stimulation and the regulation of T proliferation, we co-cultured Tregs and Tres (non-Treg) from the HDs and SSc patients with or without rIL-35. We found that the T cell proliferation was similar in both groups in cultures without rIL-35. Furthermore, the T cell proliferation was similar in both groups in cultures with rIL-35 (Figure 5A,B). In addition, the IL-17A levels on the supernatant were higher in the SSc patients than the HDs in cultures without rIL-35 (*p* < 0.01). However, the IL-17A levels on the supernatant decreased in both groups in cultures with rIL-35 (Figure 5C). The IL-10 levels on the supernatant were higher in the SSc patients than the HDs in cultures without rIL-35, while the supernatant IL-10 levels were similar in both groups in cultures with rIL-35 (Figure 5D). Moreover, the TGF-β levels were similar in both groups in cultures without rIL-35 and the TGF-β levels on the supernatant decreased in both groups in cultures with rIL-35 (*p* < 0.01) (Figure 5E).

### 2.6. Plasma Levels of IL-35 and Association with Clinical Features and Progression

**Figure 6:**
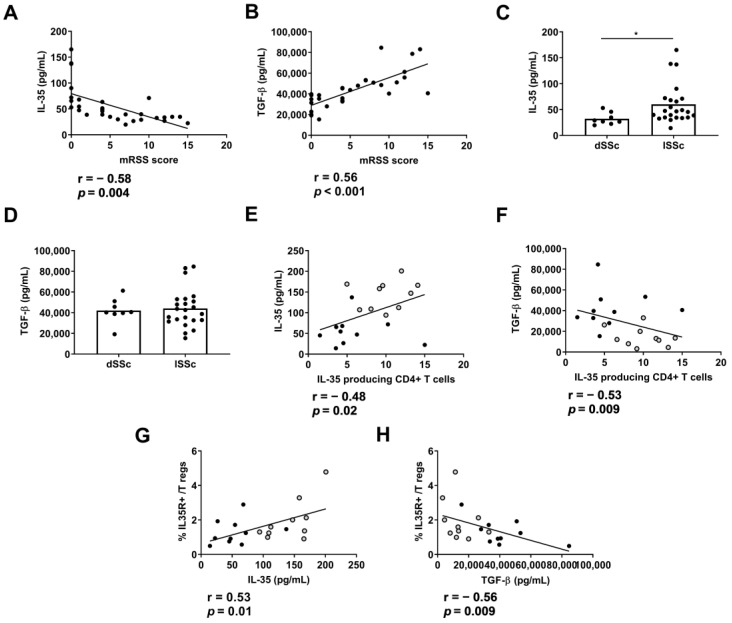
Comparison of IL-35 and TGF-β in the clinical features of SSc patients. Correlation between mRSS score and plasma levels of (**A**) IL-35 and (**B**) TGF-β in SSc patients (n = 37) (Spearman correlation). Comparison of (**C**) IL-35 and (**D**) TGF-β classified as diffuse (dSSc; n = 8) or limited (lSSc; n = 29) cutaneous SSc (Mann–Whitney U test). Correlation between the percentage of IL-35-producing CD4+ T cells and plasma levels of (**E**) IL-35 or (**F**) TGF-β (Spearman correlation). Correlation between the percentage of IL-35R on Tregs and plasma levels of (**G**) IL-35 or (**H**) TGF-β. Spots in black correspond to SSc (n = 10) patients and spots in gray to HDs (n = 10) (Spearman correlation).* *p* < 0.05.

We then analyzed the association between the plasmatic levels of cytokines and the clinical features of the patients. We found a negative correlation between the plasma IL-35 levels and skin involvement evaluated using mRSS (*p* = 0.004) (Figure 6A). In contrast, we found a positive correlation between the plasma TGF-β levels and mRSS progression score (Figure 6B). Intriguingly, the plasmatic IL-35 levels, but not TFG-b levels, were higher in the SSc patients with limited cutaneous disease (*p* < 0.05) (Figure 6C,D). We found that the percentage of IL-35+CD4+CD25+ T cells and percentage of IL-35R+ in Tregs were correlated positively with the IL-35 levels and negatively with the TGF-β levels (Figure 6E–H).

## 3. Discussion

In our study, we observed that the levels of IL-35 in plasma are lower in individuals with SSc compared to HDs. Additionally, we found a negative correlation between the plasmatic levels of IL-35 and both TGF-β and IL-17. This study represents the first evidence demonstrating a defective immunoregulatory function of IL-35 in patients with SSc. The addition of rIL-35 to SSc cells resulted in a decrease in the proliferation of CD4+ T cells, as well as a reduction in the levels of IL-17 in the cell culture supernatant and intracellular production of IL-17. However, when compared to the effects observed in the HD cells, rIL-35 did not show the ability to decrease the TGF-β levels or increase the IL-35 levels in SSc. The immunoregulatory role of IL-35 is further supported by the negative correlation observed between the intracellular production of IL-35 in CD4+ T cells and the levels of IL-17 and TGF-β in the cell culture supernatant. One potential clinical implication of our findings is that lower plasmatic levels of IL-35 are associated with greater skin fibrosis and a diffuse disease pattern.

We observed that the IL-35 concentration in the plasma was lower in the SSc patients than the HDs. This finding is similar to other rheumatic diseases, such as SLE and RA [21,22]. However, Dantas et al. reported higher serum levels of IL-35 in SSc patients compared to HDs [24]. This apparent contradiction with our results is probably due to the quantification of the IL-35 concentration at different stages of the disease. In this regard, Tomcik et al. showed that early-stage SSc patients have higher IL-35 levels than treated patients with a longer chronic disease [25]. In line with previous reports, we observed that the IL-10 and TGF-β concentrations in the plasma were higher in the SSc patients than the HDs. We also reported higher IL-17A concentrations in SSc, incremented cytokines in pulmonary fibrosis, and esophagus involvement [26,27]. Our finding of a positive correlation between TGF-β and IL-17 suggests that TGF-β increases plasmatic IL-17A levels [28]. On the other hand, the negative correlation we observed between IL-35, TGF-β, and IL-17 suggests that IL-35 suppresses the TGF-β1/Smad2/3 signaling pathway that regulates the Th17 response [29].

Despite our findings, the reason for the reduced plasmatic IL-35 levels in SSc is still unclear. One possibility is that SSc T cells cannot produce the same amount of IL-35 as HD T cells. It has been reported that IL-35 is mainly secreted by Tregs and these IL-35-producing Tregs have robust suppressive functions, inducing more IL-35 via positive feedback [18]. This is in line with Fukasawa et al., who showed that low-affinity topo I antibody-producing B cells produce inhibitory cytokines, such as IL-10 and IL-35, and were associated with inhibited fibrosis in SSc patients [30]. In addition, when we added rIL35, the IL-35+CD25+CD4+ T percentage increased in the stimulated PBMCs from HDs, but not in the PBMCs from the SSc patients. Moreover, there were fewer IL-35-producing Tregs in the active SLE patients compared to the inactive patients and healthy subjects [31]. These results indicate that the lower IL-35 production by T cells in SSc may be due to decreased IL-35 signaling and/or a reduced number of IL-35-producing CD25+CD4+ T cells.

We found that the percentage of IL-17+CD25+CD4+ T cells increased in the PBMCs from HDs and SSc patients in response to activation. However, the magnitude of this response was higher in the SSc patients. When we added rIL-35 to the activation, there was a reduction in the percentages of the IL-17+CD25+CD4+ T cells in both PBMCs. However, in this condition, we found a reduction in the TGF-β levels only in the supernatants of the activated PBMCs from the HDs. Three pieces of evidence led us to speculate that the decreased levels of IL-35 affect the Th17/Treg balance in patients with SSc and consequently favor the development of the disease. Firstly, some reports have described that IL-35 suppresses T cell effector activity and, in particular, inhibits Th17 differentiation [32,33]. Secondly, in an animal model, the secretion of IL-17A contributed to inflammation and the pathogenesis of scleroderma [4]. Thirdly, an imbalance between Th17 cells and Treg cells can trigger an autoimmune response (as shown in SLE) [34].

We also found that the activation-induced proliferation of T cells decreased in the presence of rIL-35, with subsequent reductions in the IL-17A and TGF-β supernatant levels. A similar conclusion was reached with RA patients when Nakano et al. showed that rIL-35 enhanced natural Treg function in vitro and suppressed T cell proliferation and the production of inflammatory cytokines [33]. IL-35 may promote the functional activity of Tregs through the upregulation of IL-35 receptors, which reduces T proliferation and IL-17A levels. One report showed that the co-incubation of Treg cells from HDs with plasma from SSc patients abrogated suppressive activity, suggesting the existence of factors that inhibit the Treg function in SSc patients [35]. However, the precise regulatory mechanism of IL-35 in SSc patients and how to regulate TGF-β levels require further investigation.

In concordance with previous reports, we found that skin involvement evaluated using mRSS was positively correlated with plasma TGF-β and negatively with plasma IL-35 [36]. Based on the described mechanisms in this manuscript, high levels of IL-35 likely regulate systemic inflammation, shifting toward a limited SSc development. Given that IL-35 has been reported to regulate collagen and TGF-β expression in other rheumatic diseases, IL-35 may play a protective role in SSc pathogenesis [23,32]. Plasmatic IL-35, compared to other published biomarkers, is easy to obtain via non-invasive techniques, as are its dynamics, and it can be associated with severity and classification regardless of the received treatment. However, it would be interesting to evaluate whether plasmatic IL-35 can be associated with other recent predictor markers, such as nailfold videocapillaroscopy or pharmacogenetic variant genes [37,38,39].

Despite the contribution of these results to an understanding of the role of IL-35 in SSc, our study has major limitations. First, our findings cannot be extrapolated to CD4+ T cells from tissues. Despite not being able to analyze the IL-35 expression in the tissues from these patients, other authors have suggested that it has a similar role in tissue. It has been reported that epidermal keratinocytes and Tregs express EBI3 in human skin, and that this expression is decreased in SSc patients [40]. Furthermore, this decrease in EBI3 may also contribute to the induction of IL-17 [40]. In addition, bleomycin-induced animal models have shown decreased levels of EBI3 in lung and skin fibrosis, and the supplementation of EBI3 has inhibited this fibrotic condition [40,41]. Second, we could not analyze the role of IL-35 in the monocytes from the patients. It has been reported that IL-35 is secreted by monocytes in inflammatory conditions [42]. Furthermore, increased plasmatic levels of IL-35 induce CD14+ monocytes with suppressor activity in Kawasaki disease [43]. Interestingly, IL-35 inhibition reduces cardiac recovery by decreasing monocyte/macrophage survival and the expressions of CX3CR1 and TGF-β after a myocardial infarction in mice [44]. Third, we cannot predict the function and dynamics of IL-35 in patients receiving different therapies, but we compared the effect of different treatments on the plasmatic IL-35 levels at 6 months. Fourth, an analysis of IL-35 with a larger cohort would permit a comparison of subsets of SSc patients and its potential as a potential biomarker of response.

## 4. Materials and Methods

### 4.1. Patients and Samples

A prospective observational study was performed at Hospital de la Santa Creu i Sant Pau from 2020 to 2022. The selected population was SSc patients following our scleroderma program. All of them fulfilled the ACR/EULAR 2013 systemic sclerosis classificatory criteria [45]. The patients were classified as having diffuse (dSSc) or limited (lSSc) cutaneous SSc according to LeRoy’s classification [46]. The extent of their skin involvement was assessed using the modified Rodnan skin score (mRSS) [47]. A peripheral blood sample was obtained from each patient. Written informed consent was obtained from each patient and the ethical approval for the study was granted by the Hospital de la Santa Creu i Sant Pau Institutional Ethics Committee.

### 4.2. Cell Culture and IL-35R Expression on PBMC Subpopulations

PBMCs were isolated from the heparinized peripheral blood of 37 SSc patients and 10 HDs, matched by age and sex using Leucosep tubes (Greiner Bio-One, Fricken-hausen, Germany). For the experimental analysis, we randomly selected 10 SSc patients. To evaluate the expression of IL-35R, the PBMCs were cultured in 96 well-plates (Thermo Fisher Scientific, Vienna, Austria) with RMPI-1640 (Biowest, Nuaille, France), supplemented with 10% FBS and 1% Penicillin/Streptomycin (Biowest), in the absence or presence of dynabeads human T-activator CD3/CD28 (Thermo Fisher Scientific, Waltham, MA, USA) for 72 h in 5% CO2 at 37 °C. The supernatants were collected and stored at −20 °C for cytokine determination, as described below.

**Figure 7:**
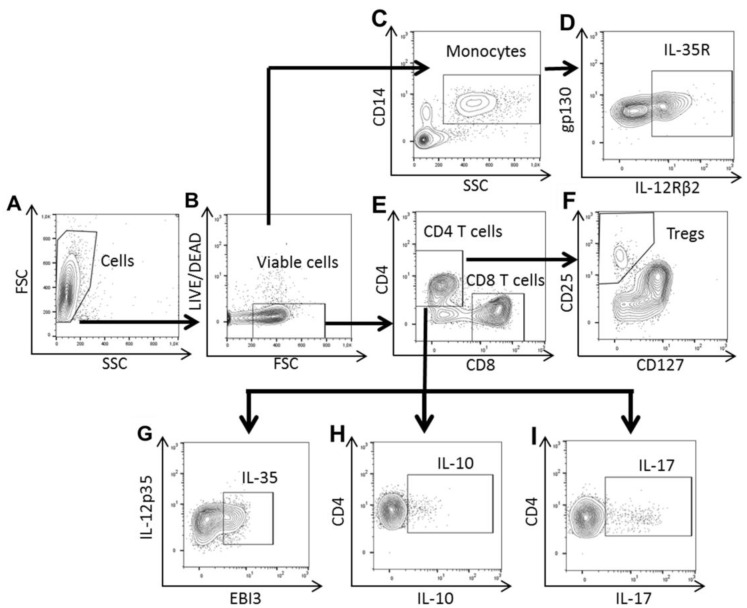
Gating strategy for the isolated PBMCs. (**A**) Lymphocytes and monocytes were selected based on their morphology using forward- versus side-scatter (FSC-SSC) dotplots. (**B**) Cells were gated with LIVE/DEAD TM Fixable Violet Dead Cell Stain Kit and FSC. (**C**) Monocytes were gated by CD14 expression and (**D**) the percentage of IL-35R+ was analyzed by combining gp130 and IL-12Rβ2. (**E**) Combining anti-CD4 and anti-CD8, we identified CD8+ and CD4+ T cells. (**F**) Tregs were identified on CD4+T cells by high CD127-CD25 expression. IL-35R was evaluated in each population as shown in D. (**G**) Percentage of intracellular IL-35 was analyzed on CD4+ T cells by combining IL-12p35 and EBI3 antibodies. (**H**) Percentages of intracellular IL-10 and (**I**) IL-17 were analyzed on CD4+ T cells.

For the IL-35R+ analysis, the PBMCs were analyzed via flow cytometry using an-ti-CD4-Viogreen (Miltenyi Biotech, Bergisch Gladbach, Germany), anti-CD3-FITC (Immunotools, Friesoythe, Germany), anti-CD127-FITC (Miltenyi Biotech), an-ti-gp130-PE (Biolegend, San Diego, CA, USA), anti-CD8 PerCP (Biolegend), an-ti-CD14-PE-Cy7 (Biolegend), anti- IL-12Rβ2-APC (Miltenyi Biotech), and an-ti-CD25-APC-Cy7 (Biolegend) antibodies (Figure 7). For the data analysis, doublets were excluded and single cells were analyzed to select lymphocytes based on their morphology using forward- versus side-scatter (FSC-SSC) dotplots. Their viability was assessed via flow cytometry using the LIVE/DEAD TM Fixable Violet Dead Cell Stain Kit (Thermo Fisher Scientific). Combining anti-CD3, anti-CD4, and anti-CD8, we identified CD8+ and CD4+ T cells, as previously reported in our laboratory [48]. Tregs were identified on the CD4+T cells by a high CD127-CD25 expression. Monocytes were gated by CD14 expression. The percentage of IL-35R+ was analyzed by combining gp130 and IL-12Rβ2 on different PBMC subsets. At least 50,000 events were acquired and the data analysis was obtained using FlowJo version 10 (FlowJo, Ashland, OR, USA).

### 4.3. Intracellular Cytokine Production by Flow Cytometry

For cytokine production, the PBMCs from HDs and SSc patients were cultured in 96 well-plates with complete RPMI 1640 and stimulated with dynabeads human T-activator CD3/CD28, in the absence or presence of 100 ng/mL of recombinant IL-35 (rIL-35; Enzo Life Sciences, Farmingdale, NY, USA) for 72 h in 5% CO_2_ at 37 °C, as previously reported in our laboratory [23]. The supernatants were collected and stored at −20 °C to determine their cytokine concentration, as described below. For the intracellular cytokine detection, the PBMCs were restimulated during the last 4 h with 50 ng/mL of phorbol myristate acetate (PMA, Sigma Aldrich, St. Louis, MO, USA) and 500 µg/mL of ionomycin (Sigma Aldrich) in the presence of 3 μg/mL of Brefeldin A (Sigma Aldrich) and Monensin (BD Biosciences, San Diego, CA, USA).

For the intracellular cytokine experiments on T cells, the PBMCs were analyzed via flow cytometry using anti-CD25-APC-Cy7 (Biolegend), anti-CD4-Viogreen (Miltenyi Biotech), anti-CD3-FITC (Immunotools), and CD8-PE-Cy7 (Biolegend) antibodies. After surface staining, the PBMCs were intracellularly stained with anti-IL-12p35-PE (eBioscience, San Diego, CA, USA), anti-EBI3-APC (BD Biosciences, San Jose, CA, USA), anti-IL-10-PE (Miltenyi Biotech), and anti-IL-17-APC (Miltenyi Biotech) on CD4+ T cells after being fixated and permeabilized with a BD Cytofix/Cytoperm kit (BD Biosciences), according to the manufacturer’s instructions (Figure 7). For the data analysis, doublets were excluded and single cells were analyzed to select lymphocytes based on their morphology using forward- versus side-scatter (FSC-SSC) dotplots. Their viability was assessed via flow cytometry using LIVE/DEAD TM Fixable Violet Dead Cell Stain Kit (Thermo Fisher Scientific). Combining anti-CD3, anti-CD4, and anti-CD8, we identified CD8+ and CD4+ T cells, as previously reported in our laboratory [48]. The percentages of intracellular cytokines in these activated T cell subsets were then analyzed. The three resulting subsets were CD3+ CD4+ CD25+IL-10+, CD3+CD4+ CD25+IL-17+, and CD3+CD4+CD25+ IL-12p35+EBI3+ for the CD4+ T cells on gated lymphocytes, as previously reported in our laboratory [23]. At least 50,000 events were acquired and the data analysis was obtained using FlowJo version 10 (FlowJo, Ashland, OR, USA).

### 4.4. Suppression Assay

For the suppression assay, the CD4+CD127+CD25+ T responder cells (Tres) and CD4+CD127-CD25+ Tregs (>95% purity) were purified using a magnetic EasySepTM Human CD4+CD127-CD25+ Regulatory T cell isolation Kit, according to the manufacturer’s instructions. Then, the Tres were incubated with 10 mM of carboxyfluorescein succinimidyl ester (CFSE; Sigma-Aldrich, St. Louis, MO, USA) and co-cultured with Tregs at a ratio of 3:1 (Tres:Treg) in the presence of dynabeads human T-activator CD3/CD28, in the presence or absence of 100 ng/mL of rIL-35. After five days of culturing, the proliferation was assessed by measuring the CFSE signals using a flow cytometry analysis. The supernatants were collected and stored at −20 °C for the cytokine determination, as described below.

The PBMCs were analyzed via flow cytometry using anti-CD25-BV421 (BD Biosciences), anti-CD4-Viogreen (Miltenyi Biotech), and CD8-PE-Cy7 (Biolegend) antibodies. The data acquisition and analysis were performed on a MACSQuant Analyzer 10 flow cytometer (Miltenyi Biotech) using FlowJo version 10. For the data analysis, doublets were excluded and single cells were analyzed to select lymphocytes based on their morphology using forward- versus side-scatter (FSC-SSC) dotplots. Their viability was assessed via flow cytometry using a LIVE/DEAD TM Fixable Violet Dead Cell Stain Kit (Thermo Fisher Scientific). We identified the Tres by combining anti-CD25, anti-CD4, and CFSE (CD25+CD4+CFSE+). At least 50,000 events were acquired and the data analysis was obtained using FlowJo version 10 (FlowJo, Ashland, OR, USA).

### 4.5. Determination of IL-35, TGF-β, IL-17A and IL-10

The plasma concentrations of IL-35 (Cloud-Clone Corp, Wuhan, China), TGF-β (Mabtech, Nacka Strand, Sweden), IL-17A (PeproTech EC, Rocky Hill, NJ, USA), and IL-10 (Immunotools) from the HDs and SSc patients were determined using specific ELISA kits according to the manufacturers’ instructions and using the specific standard curves of recombinant molecules. The limits of detection were as follows: 1.56–100 pg/mL for IL-35, 62.5–4000 pg/mL for TGF-β, 15.62–1000 pg/mL for IL-17A, and 15.62–100 pg/mL for IL-10.

### 4.6. Statistics

The statistical analyses were performed using the GraphPad Prism 7 software. The normal data distribution was performed using the Kolmogorov–Smirnov test. The variables were shown as mean ± SEM or median according to normal or non-normal distribution, respectively. The comparisons between the two groups were analyzed with the Student’s *t*-test (paired or unpaired) or the Mann–Whitney test. The comparisons of three or more groups were analyzed with a one-way analysis of variance (ANOVA) and the Bonferroni post hoc test. The correlation analyses were performed with Pearson’s or Spearman’s correlations. *p*-values of <0.05 were considered to be statistically significant.

## 5. Conclusions

Our study revealed the immunoregulatory role of IL-35 in SSc, regulating the effect of TGF-β and the inflammatory response on CD4+ T cells. These findings suggest that the plasmatic levels of IL-35 could be a potential biomarker for the diagnosis and classification of SSc patients. We are currently validating the use of the plasmatic levels of IL-35 in another cohort of patients with different therapies at different time points, with the objective of deciphering whether activating and/or expanding T cells in the presence of IL-35 may have a therapeutic role to play in the future.

## Figures and Tables

**Table 1:** Patient characteristics.

Patient Characteristics	Group	Value
Median age (range), years		65 (45–93)
Male/Female		1/31
Scleroderma phenotype, *n* (%)	Limited	8 (25%)
	Diffuse	24 (75%)
Disease duration (range), years		9 (2–32)
mRSS (mean ± SD)		5.7 ± 5.5
Skin/vascular symptoms, (%)		78.1%
Renal symptoms, (%)		0%
Gastrointestinal symptoms, (%)		59.4%
Pulmonary symptoms, (%)		18.8%
Treatment, (%)	Immunosupressors	37.5%
	Biological therapy	21.9%
	Steroids	37.5%

## Data Availability

All data relevant to the study are included in the article or The datasets used and analyzed during the current study are included in the article. They are available from the corresponding author on reasonable request.
